# Special Issue: Application of Nanomaterials in Biomedical Imaging and Cancer Therapy

**DOI:** 10.3390/nano12050726

**Published:** 2022-02-22

**Authors:** James C. L. Chow

**Affiliations:** 1 Radiation Medicine Program, Princess Margaret Cancer Centre, University Health Network, Toronto, ON M5G 1X6, Canada; james.chow@rmp.uhn.ca; 2 Department of Radiation Oncology, University of Toronto, Toronto, ON M5T 1P5, Canada

Nanomaterials of different types—namely, inorganic-based, organic-based, carbon-based, and composite-based ones, with various structures such as nanoparticles, nanofibers, nanorods, nanoshells, and nanostars, all have demonstrated a wide range of medical biophysical and chemical properties. Taking advantage of recent advanced synthetic and drug delivery techniques, nanomaterials become superior candidates for a wide range of usages, including dose enhancers, contrast agents, and drug delivery vehicles in biomedical imaging and cancer therapy. To celebrate the rapid application development of nanomaterials in biomedical imaging and cancer therapy, this Special Issue entitled “Application of Nanomaterials in Biomedical Imaging and Cancer Therapy” is an innovative collection of nanomaterial studies on various types of cancer therapy such as hyperthermia, radiotherapy, immunotherapy, photothermal therapy, and photodynamic therapy, as well as medical imaging such as high-contrast and deep-tissue imaging, quantum sensing, super-resolution microscopy, and three-dimensional correlative light and electron microscopy.

This Special Issue begins with a review of nanomaterial applications in biomedical imaging and cancer therapy [1]. This topical review focuses on the latest studies of nanotechnology on theranostic applications and explores the role of nanomaterials in biomedical imaging and cancer therapy. Studying hyperthermia, Caizer carried out a computational study on the magnetic properties of Co_x_Fe_3−x_O_4_ ferrite nanoparticles used in superparamagnetic hyperthermia [2]. Through computational simulations on the Co_x_^2+^ ion concentration with x = 0 to 1, the efficiency of hyperthermia of tumours can be optimized to maximum using the Co_x_Fe_3−x_O_4_ nanoparticles. Focusing on radiotherapy, Jabeen et al. performed a Monte Carlo simulation to investigate the DNA dosimetry and damage when photon beams irradiated a DNA molecule with a gold nanoparticle under a magnetic field [3]. The simulation results provided significant information regarding dose enhancement at the DNA in gold nanoparticle-enhanced radiotherapy, using magnetic resonance imaging-guided linear accelerator. Examining immunotherapy, Yan et al. provided a comprehensive review on the pH-responsive nanoparticles in cancer treatment [4]. The review investigated the challenges of immunotherapy on inadequate efficacy and toxic side effects. They concluded that using pH-responsive nanoparticles in immunotherapy could manage these challenges. This is because the nanoparticles can target tumour tissues and organelles of antigen-presenting cells, which have an acidic microenvironment.

Pivetta et al. reviewed the nanoparticle systems for photodynamic and photothermal therapy [5]. They highlighted the technical developments of drug encapsulation and surfaces’ functionalization of organic- and inorganic-based nanoparticles to improve the effectiveness of phototherapy. In photothermal therapy, spiky gold nanoparticles were synthesized by carefully controlling the nanoparticle growth process [6]. These branched gold nanoparticles demonstrated large absorption in the first near-infrared (NIR) window and efficient light-to-heat conversion capability under 880 nm lasers. It is, therefore, possible to use these gold nanoparticles in photothermal therapy for colon cancer. At the same time, Zhao et al. developed organic molecular-based nanoparticles, which were novel 880 nm NIR laser-triggered agents for photothermal therapy of tumour [7]. The in vitro results showed that these organic-based nanoparticles had superior biocompatibility and phototoxicity. Moreover, the nanoparticles exhibited good photothermal stability and conversion efficiency. To treat lung cancer using photothermal therapy, plasmonic gold nanorods were developed and functionalized with anti-EGFR antibodies [8]. The performance of nanorods was tested by the pulsed lasers and continuous-wave lasers based on lung cancer cells (A549). The in vitro results demonstrated that the combination of pulse wave laser illumination of nanorods could produce about a 93% reduction in cell viability, compared with control exposures. In studying drug delivery, Marpu et al. proposed a one-step method for the photochemical formation of NIR-absorbing gold nanomosaic using thermoresponsive poly(N-isopropylacrylamide) microgels [9]. This nanomosaic was generated based on photochemical reduction of a gold precursor, without reducing agent or growth-assisting surfactants. In a drug release model, photothermal shrinkage in microgels was shown by the release of a model luminescent dye.

For biomedical imaging, a silica coating method was reported for upconversion nanoparticles that did not agglomerate post-annealing [10]. These photostable, YVO_4_:Yb,Er nanoparticles produced bright visible emission reacting to NIR light and had potential in many imaging applications such as quantum sensing, super-resolution microscopy, and deep-tissue imaging. Furthermore, Prabhakar et al. proposed a method for executing three-dimensional correlative light and electron microscopy using fluorescent nanodiamonds as multimodal probes [11]. Nanodiamonds are fluorescent, nanosized, and electron-dense materials, which are biocompatible and easily identified in living cell fluorescence imaging and serial block-face scanning electron microscopy.

This Special Issue covered a wide spectrum of most recent nanomaterial applications in biomedical imaging and cancer therapy. The results and findings in this issue are expected to be useful for researchers who are working in medical nanotechnology. Finally, I would like to express my heartfelt gratitude to all authors who contributed their innovative research to this Special Issue.
